# Deep-Subwavelength-Optimized Holey-Structured Metamaterial Lens for Nonlinear Air-Coupled Ultrasonic Imaging

**DOI:** 10.3390/s21041170

**Published:** 2021-02-07

**Authors:** Marco Boccaccio, Pasquale Rachiglia, Gian Piero Malfense Fierro, Giovanni Pio Pucillo, Michele Meo

**Affiliations:** 1 Department of Mechanical Engineering, University of Bath, Claverton Down, Bath BA2 7AY, UK; m.boccaccio@bath.ac.uk (M.B.); pasqualerachiglia@gmail.com (P.R.); gpmf21@bath.ac.uk (G.P.M.F.); 2 Department of Industrial Engineering, University of Naples Federico II, 80125 Naples, Italy; gpucillo@unina.it

**Keywords:** metamaterial, holey lens, nonlinear ultrasonics, deep-subwavelength, air-coupled, non-destructive testing, acoustics

## Abstract

Ultrasound non-destructive testing (NDT) is a common technique used for defect detection in different materials, from aluminium to carbon-fiber-reinforced polymers (CFRPs). In most cases, a liquid coupling medium/immersion of the inspected component is required to maximize impedance matching, limiting the size of the structure and materials. Air-coupled inspection methods have recently been developed for noncontact inspections to reduce contact issues in standard ultrasonic inspections. However, transmission of ultrasound in air is very inefficient because of the enormous impedance mismatch between solids and air, thus requiring a signal amplification system of high-sensitivity transducers. Hence, the captured signal amplitude may not be high enough to reveal any wave distortion due to defects or damage. This work presents a design of a holey-structured metamaterial lens with a feature size of λ/14 aiming at improvement of acousto-ultrasonic imaging using air-coupled transducers. The required effect is obtained by matching geometrical parameters of the proposed holey-structured metamaterials and the Fabry–Perot resonance modes of the structure. Transmission tests have been conducted on different fabricated metamaterial-based structures, to assess the frequency component filtering of the proposed method in both acoustic (f = 5 kHz, 20 kHz) and ultrasonic range (f = 30 kHz, 40 kHz). Results showed an improved sensitivity of damage imaging, with an increase in amplitude of the design frequencies of the lens by 11 dB. Air-coupled inspections were conducted on a stress-corrosion cracked aluminum plate and impacted CFRP plate using the holey-structured lens. Results showed an improvement in the damage-imaging resolution due to a wave-amplitude increase across the defective features, thus demonstrating its potential as an efficient and sensitive inspection tool for damage-detection improvement in geometrically complex components of different materials.

## 1. Introduction

Structural health monitoring (SHM) and non-destructive testing (NDT) methods are of interest in many engineering fields, such as railway, automotive and aerospace, to provide quantitative information about structural properties, integrity and residual life of the test-pieces being inspected [1,2,3,4]. Non-invasive testing on components leads to a reduction in maintenance costs and improvements in safety. The most common inspection techniques employed in industry typically rely on the use of magnetic particles (flaws visualization), X-ray, ultrasound or eddy currents (scanning inspection). Among other NDT techniques, ultrasound has gained research momentum owing to its safety in terms of radiation risks, affordability and capability to detect internal damage. State-of-the-art techniques, such as phased array ultrasound, use multiple sensor/actuators to interrogate defects in materials, although these methods generally require coupling mediums, in order to remove the air gap between the probe and sample [5,6,7]. However, the efficiency of conventional ultrasound methods strongly depends on the size of the defects and the ability to capture distortions in reflected or transmitted signals. In addition, these ultrasound-based techniques suffer from an inability to find micro-damages and unacceptable resolution, limited by the classical Rayleigh reflection limit to half wavelength [8,9]. Ultrasonic excitation at higher frequencies (i.e., MHz and GHz) can provide higher resolution, but this results in very poor penetration into the inspected component. Air-coupled ultrasound (ACU) methods provide noncontact inspections in which coupling media are not appropriate, e.g., in such industrial processes in which immersion or contact techniques may not always be practical, or in which water could damage the material, such as wood, reinforced plastics and electronic-packaging material [10,11,12,13]. However, the main issue facing the mainstream adoption of these methods is represented by high losses in energy, due to a massive acoustic mismatch between air and metals, resulting in very low transmitted energy. Several strategies have been adopted to overcome these limitations. Indeed, an acoustic matching layer for improving energy transfer into the test piece has been adopted for ACU inspection [14,15,16], but the efficiency of the employed materials is restricted in an unacceptable narrow bandwidth. Moreover, the sound energy can be enhanced by exciting the sensor with a high-voltage signal by means of amplification systems, but both the restrained voltage range of the transducers and a poor signal-to-noise ratio represent drawbacks of these techniques [17]. Several strategies involving a pulse-compression technique have been developed to improve the dynamic range in the ultrasonic air-coupled technique, by taking advantage of a particular auto-correlation feature of the Golay code [18,19,20], in order to improve ultrasound SNR. However, the Golay method for impulse-response measurement has been found to be susceptible to measurement artefacts due to time-varying systems, which can affect the performance of the inspections [19]. Although ACU issues have been addressed, further improvements are needed. This works aims at solving such issues by using acoustic metamaterial structures to improve the amplitude of the captured signal in air-coupled inspections. In recent years, the metamaterials field has gained research momentum owing to its ability to focus acoustic waves based on the refractive and geometric properties of the structure. The majority of the developed acoustic metamaterials lenses are based on the principle of negative refractive index [21,22,23,24,25]. These artificial structures have been found to provide better resolution above the diffraction limits, while having serious practical and manufacturing limitations. Russel et al. [26] provided a dual-core preform optical modulator for resonance localisation at megahertz (MHz) frequencies of sonic band gaps in crystal fibre preforms, aimed at increasing sensing to detect damages. However, the frequency range is not suitable for air-coupled inspections in most engineering fields or in real applications, where the operating frequency range is in the kilohertz (kHz) range.

In this work, an acoustic metamaterial lens was designed to increase the amplitude of an air-coupled NDT inspection based on the principle of holey-structured metamaterials [27,28]. We describe the detailed evaluation of optimal geometrical parameters of the lens to couple the driving frequency of the inspection with the resonance mode of the holey-structured lens. Finally, the optimized lens was designed and printed, in order to perform air-coupled inspections on different components and demonstrate its potential and versatility in deep-subwavelength acousto-ultrasonic imaging of defects of various scales and natures in different materials.

## 2. Theoretical Background

### 2.1. Air-Coupled Ultrasound

**Figure 1:**
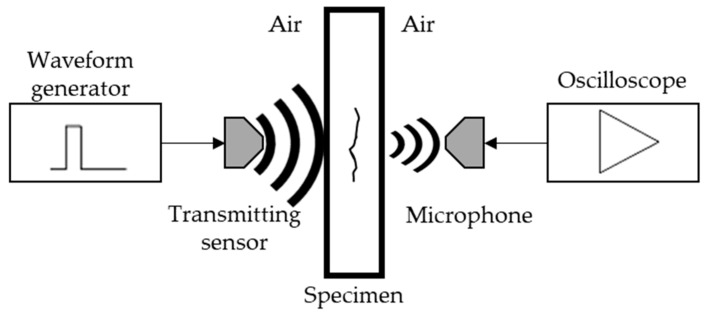
Schematic representation of an air-coupled ultrasound (ACU) system.

**Figure 2:**
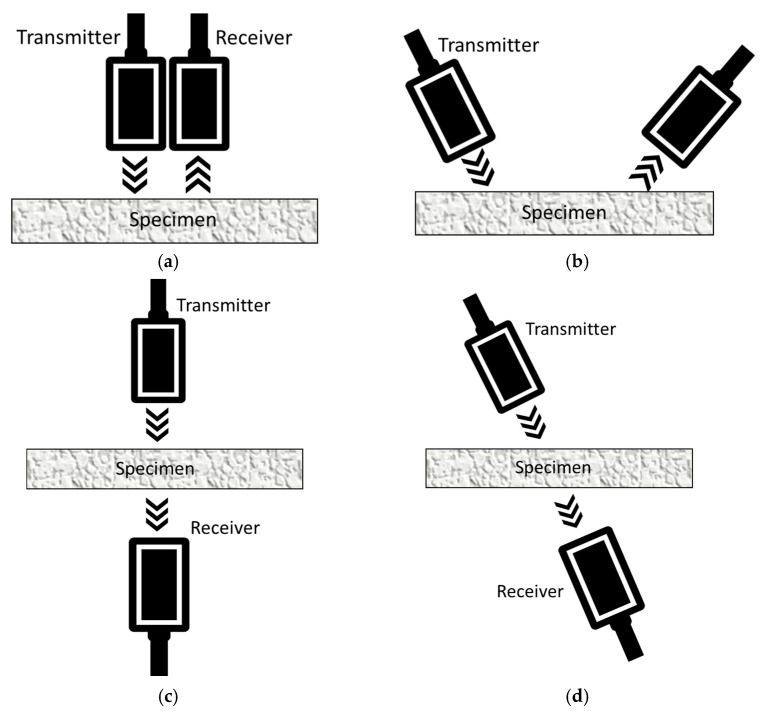
Transducer alignment in different excitation-capturing models: (**a**) pulse-echo; (**b**) plate wave; (**c**) perpendicular through-transmission; (**d**) oblique through-transmission.

In recent decades, air-coupled ultrasound (ACU) inspections have found wide applications in many engineering fields for material characterization and damage detection. These techniques are used to perform detailed noncontact inspections to detect cracks in materials that are not compatible with a coupling medium (Figure 1). First employed in the aerospace industry in composite materials, they found applications in a wide range of materials in which conventional NDT techniques involving contact are not feasible (foams, fuel grain and corrosion) [29,30,31]. ACU inspections are commonly used in single-point detection systems for flat sample testing. Indeed, an air-coupled device can be also installed in existing equipment to inspect large components, thanks to a simple setup, and provide similar results compared with conventional contact techniques. In this regard, ACU sensors can be aligned in different modes to excite and capture waves, including pulse-echo, plate wave, perpendicular through-transmission or oblique through-transmission, according to the accessibility and the working condition of the component being inspected. The different sensor arrangements are illustrated in Figure 2.

Ultrasonic wave propagation in air has generally received major attention. Indeed, the main limitation of this technique relies on the large acoustic impedance mismatch between solids and fluid, as shown in Table 1. In an ACU system, the captured signal amplitude is mainly affected by the transmission occurring at fluid–solid interfaces within the system (i.e., transducer–fluid and fluid–specimen). In addition, smaller-scale losses can be also considered, due to diffraction, finite-amplitude saturation effects and loss of phase-front coherence, which generally occur at very high drive levels [17].

The signal-to-noise ratio (SNR) in an ACU system is mainly related to instrumentation and the inspected material, and can be determined as follows:

(1) $$\mathrm{SNR}=10\log\left[\left(\frac{T}{F\alpha_{f}\alpha_{s}C_{L}}\right)\frac{V^{2}}{16Rk\tau \Delta f}\right]$$

where *T* is the pressure transmission coefficient at the fluid-solid interface, and *F* represents the noise resistance, which depends on the transducer and its effectiveness to increase the electrical input resistance to the resistance level related to the equivalent noise resistance of the amplification system employed. $\alpha_{f}$ and $\alpha_{s}$ are the sound absorption in air and in the solid, respectively; $C_{L}$ consider the “two-way” transducer transmission loss; *V* represents the peak-to-peak excitation voltage; *R* is the loading resistance of the amplification system; *k* is Boltzmann’s constant; τ denotes the absolute temperature; and Δf is the effective frequency bandwidth of the receiver electronics. Assuming ultrasonic waves propagating at the interface between two media 1 and 2 at angle of incidence $\theta_{i}$ and resulting transmitted angle $\theta_{t}$ (e.g., Snell’s law [32]), the corresponding pressure reflection R and transmission T coefficient are given as:

(2) $$R=\frac{Z_{2}\cos\theta_{t}-Z_{1}\cos\theta_{i}}{Z_{2}\cos\theta_{t}+Z_{1}\cos\theta_{i}};\;T=1-R=\frac{2Z_{2}\cos\theta_{i}}{Z_{2}\cos\theta_{t}-Z_{1}\cos\theta_{i}}$$

In accordance with the values in Table 1, T is approximately 10^−8^ at the interface between aluminum (Z_air_ = 430 Rayls) and air (Z_Al_ = 17.1 MRayls), thus resulting in an 80 dB amplitude loss (i.e., 99.7% of the signal being reflected back from the interface). This can be challenging for conducting a nonlinear air-coupled inspection and for detecting damage and flaws, where a high-sensitivity transducer or a high-power amplification system is required. To compensate for this energy loss, we present the design of holey metamaterial lenses to enhance the amplitude of the received signals.

### 2.2. The Fabry–Perot Resonance Modes—Principle of Metamaterial Lenses

Holey metamaterial lenses consist of a rigid structure with deep-subwavelength periodical squared cavities of periodicity *Γ* that gain particular acoustic properties from their geometrical properties and spacing. Resonance is achieved in accordance with the size of the lens, in terms of length *l*, hole size *d* and periodicity of the cavities. In the holey metamaterial lens, each cavity behaves as a pixel for enhanced near-field imaging by increasing the amplitude of the decaying evanescent waves through Fabry–Perot transmission resonance [27,33,34]. For deep-subwavelength imaging, the evanescent waves reflected from the structure can be used to excite the Fabry–Perot resonance mode, resulting in a full transmission of the evanescent waves through the metamaterial lens. Since such waves carry more wave vectors than that of the propagating wave inside the cavities, they can be used to restore images with a feature size far below the diffraction limit. Moreover, the frequency-excitation mechanism can be explained within the effective medium approach and wave equation. Assuming an acoustic plane wave scattered by the front aperture of the holey structure and propagating through the cavities in the limit in which all diffraction effects are neglectable and the transmission occurs through the fundamental propagating mode inside the cavities, (i.e., *λ >> d*, *Γ* where *λ* is the wavelength) [35], the wave equation can be written as follows:

(3) $$\left(\nabla^{2}+k^{2}\right)p=0$$

where *k* is the wave number and *p* is the acoustic pressure. Furthermore, the transmission coefficient for a plane wave propagating with parallel momentum $k_{p}=\sqrt{k_{x}^{2}+x_{y}^{2}}$ is given below [36]:

(4) $$T=\;\frac{4{\left|\frac{d}{\Gamma }\right|}^{2}X_{k}e^{\left(ik_{o}l\right)}}{{\left(1+X_{k}{\left|\frac{d}{\Gamma }\right|}^{2}\right)}^{2}-X_{k}{\left(1-{\left|\frac{d}{\Gamma }\right|}^{2}\right)}^{2}e^{\left(i2k_{o}l\right)}}$$

where *k*_0_ is the propagation constant of the fundamental propagating mode within the cavities, and *X_k_* is the ratio between the wavenumber and parallel momentum. Since the metamaterial walls are rigid and due to the acoustic impedance mismatch, the wave does not propagate into the solid parts of the structure. As a result, the lens–air interface can be assumed as rigid (i.e., $\partial p/\partial n=0_{\;}$). Consequently, the transmission coefficient has a unit modulus for all the parallel directions (including evanescent waves) when the resonance condition for standing-wave generation is satisfied (i.e., $q_{z}h=m\pi$, where m is an integer). The unique solution in terms of length *L* satisfying the standing-wave condition leading to the Fabry–Perot resonant mode (i.e., *T* = 1 and *m* = 1) is given by:

(5) $$L=\frac{\lambda }{2}$$

where *m* is an integer. Considering an acoustic wave reflected from the front aperture of the holey structure and propagating inside the cavities in such a way to neglect all the diffraction effects, and that wave transmission relies on the fundamental propagating mode inside the cavities, the hole size d must be much smaller than the wavelength *λ* [35,36,37]:

(6) d=λn λ≫d

**Figure 3:**
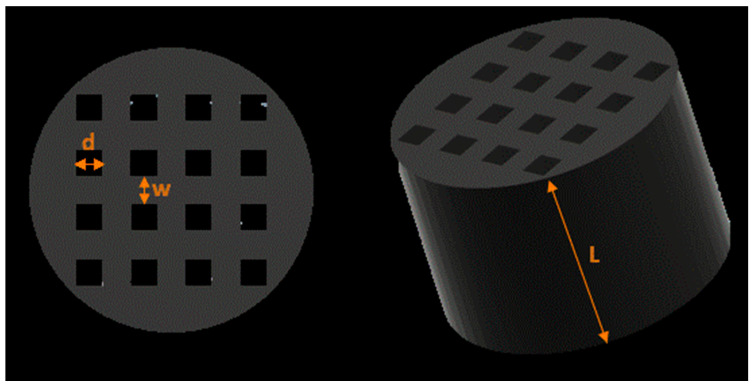
The holey metamaterial lens. Dimensions: d is hole size, w represents the spacing between two walls (i.e., wall thickness), and L denotes the length of the cavities.

An acceptable condition can result in the scenario where λ is an order of magnitude higher than d (i.e., n≥10). The expression for the transmission coefficient in Equation (4) can be approximated as a sum of multiple reflection in the holes, thus:

(7) $$T=\;\frac{\theta_{12}\theta_{23}e^{\left(ik_{o}l\right)}}{1-\sigma^{2}e^{\left(i2k_{o}l\right)}}$$

where $\theta_{12}=2\left(d/\Gamma \right)/\left[1+X_{k}{\left(d/\Gamma \right)}^{2}\right]$ is the transmission amplitude of the incident plane wave coupled with the fundamental propagating mode within holes; $\theta_{23}=2X_{k}\left(d/\Gamma \right)/\left[1+X_{k}{\left(d/\Gamma \right)}^{2}\right]$ represents the transmission amplitude by which the wave guide, as travelling against the interface, propagates into the zero-order diffraction beam in the transmission region; and $\sigma =[X_{k}{\left(d/\Gamma \right)}^{2}-1]/[X_{k}{\left(d/\Gamma \right)}^{2}+1]$ denotes the reflected amplitude of the fundamental mode inside the hole. The most relevant scenario occurs when the coupling between the outgoing propagating plane wave and the fundamental mode inside the hole becomes weak (i.e., $\left|\theta_{23}\right|\ll 1)$ [36]. This results in a deep-subwavelength region (σ=1), where the two interfaces of the cavities act as quasi-perfect sound-wave mirrors. In this scenario, $\left|\theta_{23}\right|\propto 2\left(d/\Gamma \right)$ or Γ∝2d, hence the periodicity Γ is required to be proportional to twice the hole size for perfect transmission. According to Figure 3, the periodicity Γ can be considered as the sum of the hole size and wall thickness, as follows:

(8) Γ=d+w

According to Equations (6) and (8), and with the proportionality of the periodicity Γ with the hole size d, the optimum wall thickness is given by:

(9) $$w=\Gamma -d=\frac{\lambda \left(2p-1\right)}{n}$$

where *p* is an integer.

## 3. Materials and Methods

Various holey metamaterials lenses were designed according to Equations (5)–(9) in order to assess the performance of the developed approach in both the acoustic and ultrasonic ranges; the dimensions of the developed structures are summarized in Table 2. Figure 3 shows the meanings of the geometrical parameters used to realize the holey lens structure according to the above-mentioned theory.

**Figure 4:**
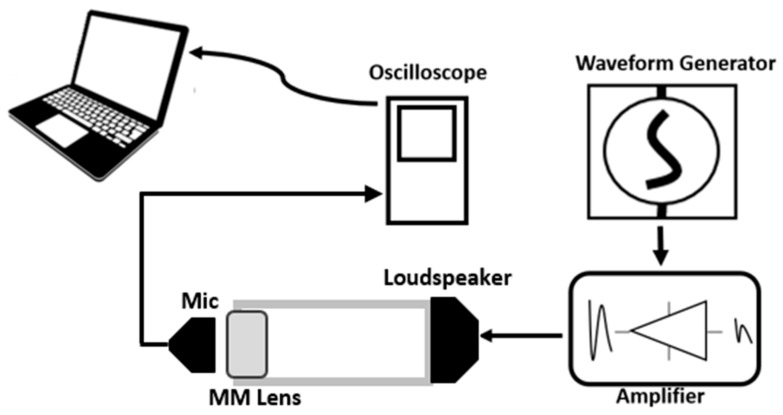
Graphic representation of experimental set-up. A waveform generator was used to send the wave inside the tube via the loudspeaker. A signal power amplifier was also employed to amplify the input signal’s amplitude. The holey lens was placed on the other opening of the tube, while a receiving sensor was positioned outside the tube to acquire the propagating wave.

**Figure 5:**
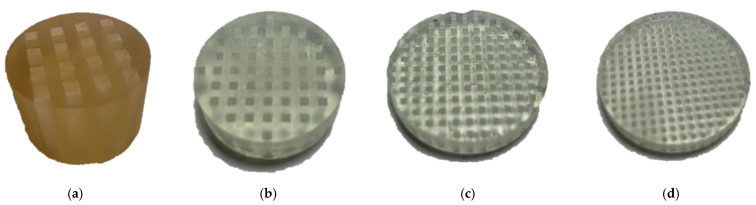
3D-printed holey structures:(**a**) structure S1 (5 kHz); (**b**) structure S2 (20 kHz); (**c**) structure S3 (30 kHz); (**d**) structure S4 (40 kHz).

**Figure 6:**
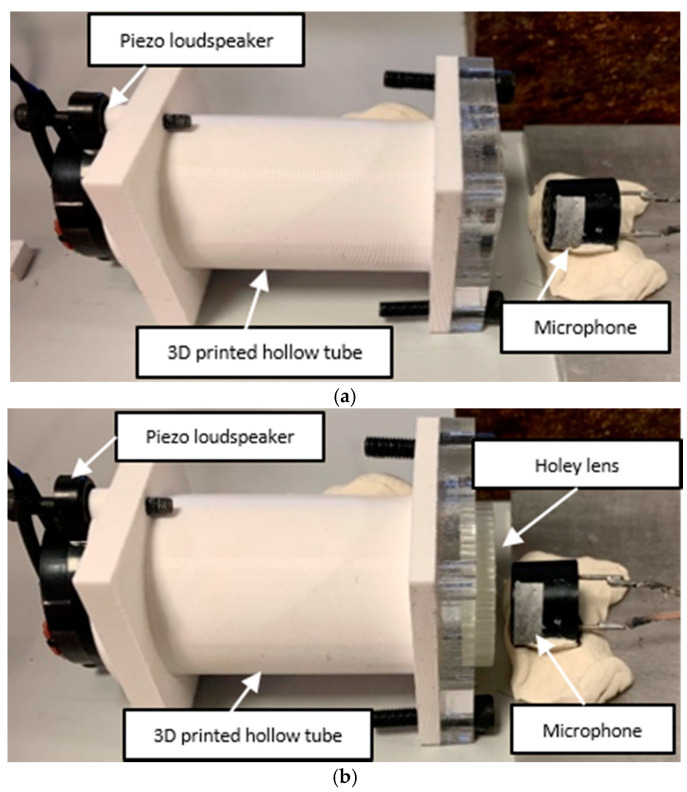
Experimental set-up for transmission tests. The 3D-printed hollow tube had a piezo loudspeaker on one side and a free opening on the other side. (**a**) A microphone was placed outside the free opening to capture propagating wave without the holey structure. (**b**) To test the holey structure; the holey lens was placed at the free side, and the microphone was positioned behind the structure.

Transmission tests were performed in order to assess the effectiveness of the designed holey structures. The equipment used in the inspections was composed of a miniature piezo loudspeaker 12–24 V, frequency bandwidth 25–45,000 Hz, and a receiving ultrasonic sensor, 16 mm diameter, 40 kHz, −65 dB, −30 °C to 80°. The experimental setup is illustrated in Figure 4. An acrylonitrile butadiene styrene (ABS) 3D-printed hollow tube was used to conduct the inspection, during which the loudspeaker was placed at one side with the holey lens on the opposite opening, while the receiving sensor was positioned behind the lens outside the tube. A dual-channel Function/Arbitrary TTi TG501140 50 MHz arbitrary waveform generator was used to generate the signal through the loudspeaker, in conjunction with a Ciprian (Saint Ismier, France) US-TXP-3 50× high-voltage power amplifier, which amplified the input signal subjected to attenuation due to the energy losses in air. An oscilloscope was linked to the receiving sensor to sample the response. The acquired signal was then post-processed via MATLAB algorithms. The holey-structured lenses were fabricated via photoresin-based stereolithography (SLA) 3D-printing technology [38,39,40], in which the production of the object relies on the solidification of a liquid resin by photopolymerization performed with a focused laser (Figure 5). Tests were performed with and without the holey-structured metamaterial lens, as shown in Figure 6.

**Figure 7:**
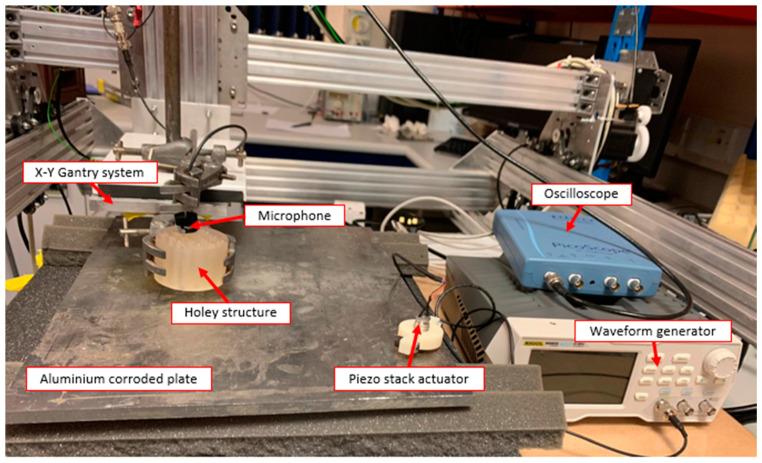
Experimental set-up of the plate scans.

**Figure 8:**
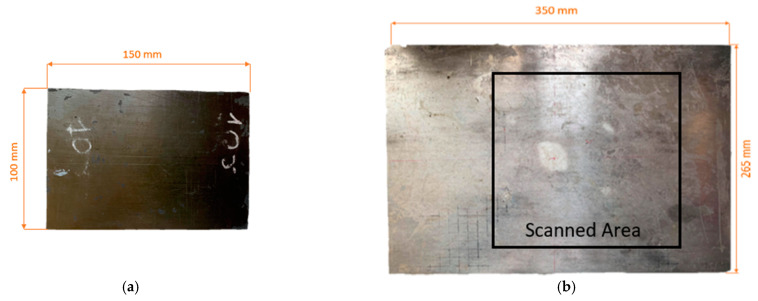
Inspected samples: (**a**) the carbon-fiber-reinforced (CFRP) polymer 100 × 150 × 4 mm plate, subjected to a 10 J impact loading in the center, showing barely visible impact damage (BVID); (**b**) 350 × 265 × 8 mm aluminium plate with induced stress-corrosion cracks in the centre.

**Figure 9:**
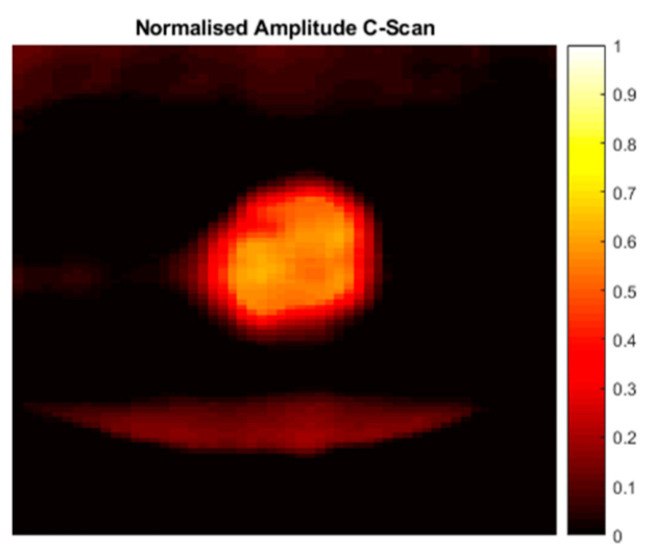
PA C-Scan imaging of the impacted CFRP plate.

Furthermore, the holey metamaterial-based structure principle was applied to an ultrasound air-coupled NDT technique, to improve nonlinear damage imaging. As shown in Figure 7, an ultrasonic system composed of a piezo-stack actuator (central frequency 76 kHz) attached to the sample to induce mechanical vibration at high frequency, and a microphone to acquire the response of the excited structure, was employed to conduct a noncontact inspection of the surface of the test pieces. The developed lens was positioned close to the surface, with an air gap of 20 mm, while the microphone was placed on the opposite side of the lens, and moved along the surface of the sample by means of a X-Y 2 mm step-gantry system. Then, an oscilloscope was employed to acquire the response. Finally, the received signals were then post-processed via LabView and MATLAB algorithms, where the amplitude of the driving frequency at each point was evaluated. These inspections were conducted on a 100 × 150 mm carbon-fiber-reinforced polymer (CFRP) plate (Figure 8a) damaged by a low-velocity instrumented impact at 10 J (the C-scan damage visualization by means of phased-array ultrasonic techniques (PAUTs) [7,41] is shown in Figure 9), and an aluminum plate 350 × 265 mm (Figure 8b) with atmospherically induced stress-corrosion cracks. The corrosion damage was introduced by putting droplets of seawater and magnesium chloride (MgCl_2_) on the plate. An additional test was conducted on the aluminum stress-corrosion cracked plate without the metamaterials, to compare damage evaluation results by using the proposed metamaterial-based holey structure.

## 4. Results and Discussion

Transmission tests were performed on each holey structure by exciting the structure with a single-frequency continuous sine wave. The captured time-domain signals were sampled and processed in MATLAB, where a fast Fourier transform (FFT) was applied to visualise the frequency response of the system. The responses with and without holey structures were then compared for each frequency range.

**Figure 10:**
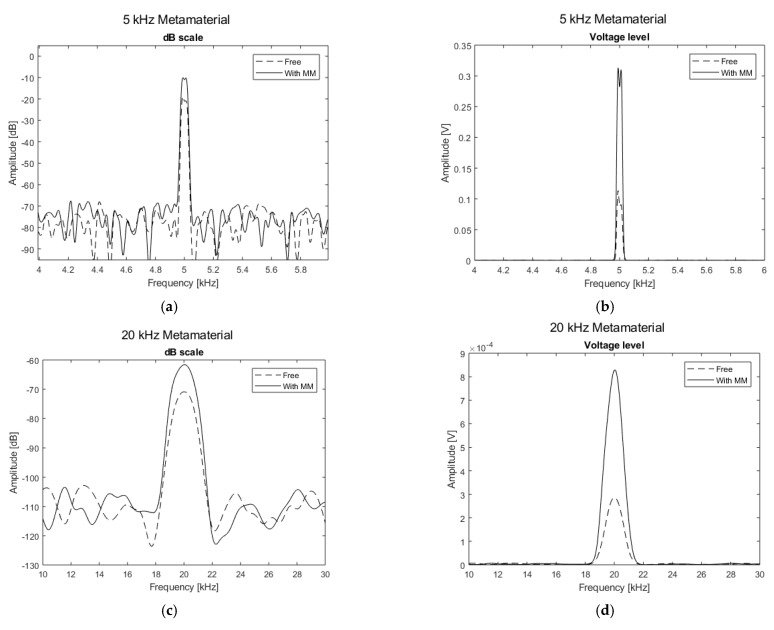

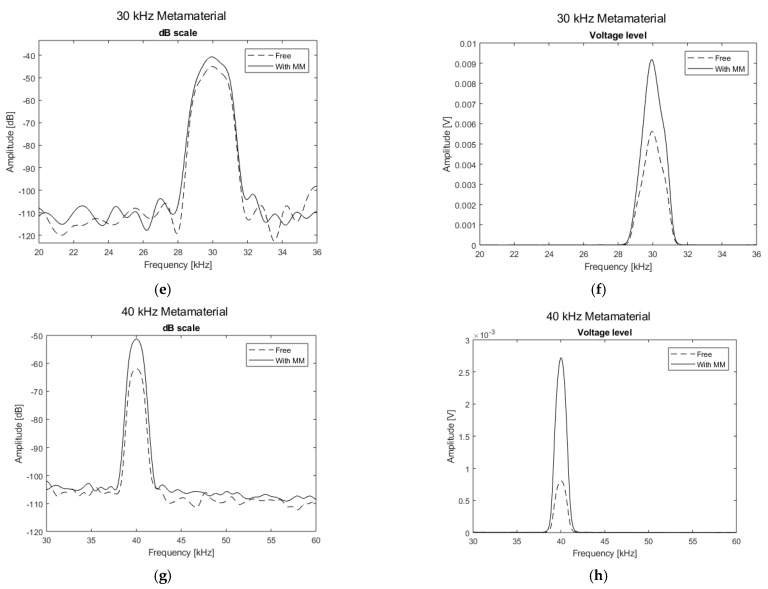
Transmission test results showing frequency spectra of the acquired signals: (**a**) S1 (f = 5 kHz) in dB scale, amplitude gain +11 dB; (**b**) S1 (f = 5 kHz) in voltage scale (+254% of the wave amplitude); (**c**) S2 (f = 20 kHz), amplitude gain +9 dB; (**d**) S2 (f = 20 kHz), in voltage scale (+192% of the wave amplitude); (**e**) S3 (f = 30 kHz) in dB scale, amplitude gain +5 dB; (**f**) S3 (f = 30 kHz) in voltage scale (+64% of the wave amplitude); (**g**) S4 (f = 40 kHz) in dB scale, amplitude gain +8 dB; (**h**) S4 (f = 40 kHz) in voltage scale (+145% of the wave amplitude).

**Figure 11:**
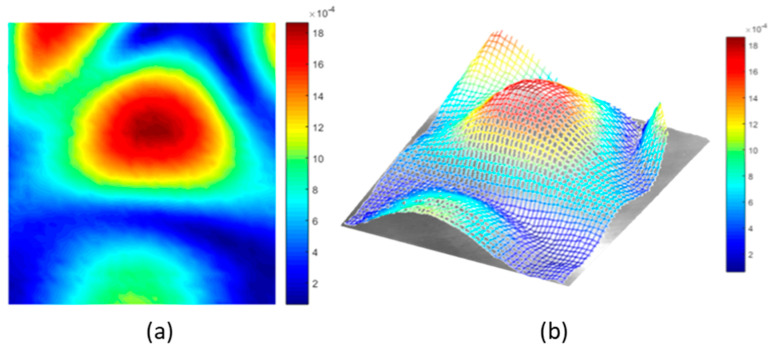
Air-coupled results on an impacted CFRP plate. Inspection conducted with the 5 kHz holey-structured metamaterial—amplitude of the excitation frequency: (**a**) surface plot; (**b**) 3D surface plot.

**Figure 12:**
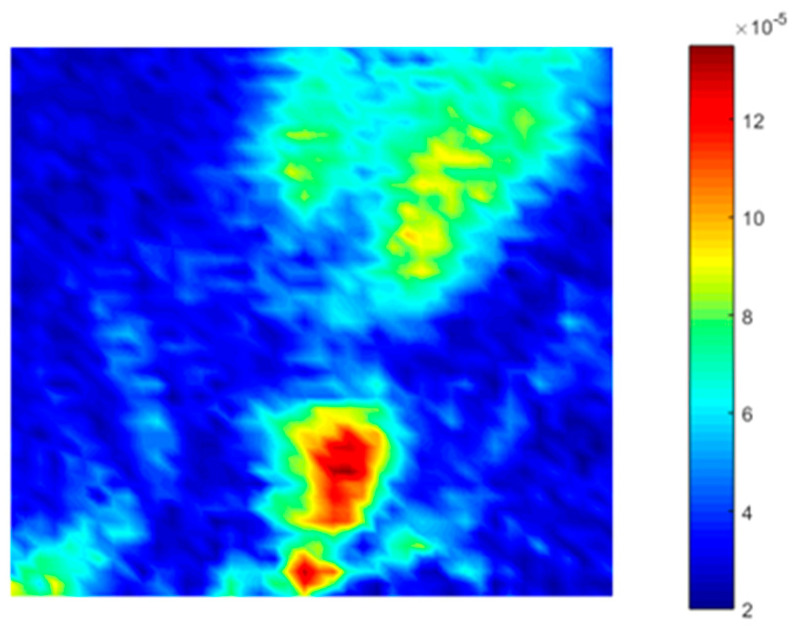
Air-coupled results on the aluminium stress-corrosion cracked plate, inspection conducted without metamaterial—amplitude plot of the excitation frequency.

**Figure 13:**
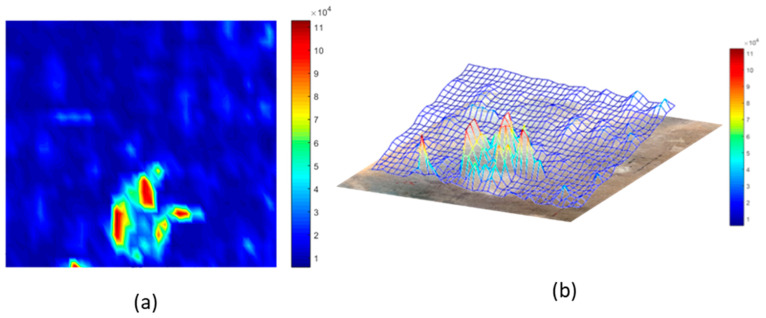
Air-coupled results on the stress-corrosion cracked plate. Inspection conducted with the 5 kHz holey-structured metamaterial—amplitude plot of the excitation frequency: (**a**) surface plot; (**b**) 3D surface plot.

For structure S1 (see Table 2), a 5 kHz 5 V peak-to-peak continuous sine wave was used as excitation. Single-frequency test results (Figure 10b) showed an increase of 254% (+11 dB in Figure 10a) in the amplitude of the corresponding design frequency (5 kHz) when the holey structure was placed between the miniature piezo loudspeaker source and the receiving ultrasonic sensor. For structure S2 (design frequency 20 kHz) a 20 kHz 5 V peak-to-peak continuous sine wave was used to perform transmission tests. The response with the holey-structured metamaterial lens showed an increase of 192% (+9 dB in Figure 10c) in the amplitude compared to the response without the structure corresponding to 20 kHz (i.e., the design frequency of the S2), as shown in Figure 10d.Structure S3 was excited by sending a 30 kHz 5 V peak-to-peak continuous sine wave. Figure 10f illustrates the single-frequency test results for structure S3, with an increase of 64% (+5 dB in Figure 10e) in the amplitude of the design structure frequency (30 kHz) when the holey structure was placed between the sensors. Finally, for structure S4, a 40 kHz 5 V peak-to-peak continuous sine wave was used as the excitation signal. The single-frequency test results (Figure 10h) showed an increase of 145% (+8 dB in Figure 10g) in amplitude of the corresponding design frequency (i.e., 40 kHz) when the holey structure was positioned between the sensors. Results related to the inspection performed along the impacted CFRP plate and corroded aluminium plate are shown in Figure 11 and Figure 13, respectively. Both the microphone and the holey-structured metamaterials were moved around the surface of the samples by means of an X-Y gantry system (step size 2 mm). Collected data were then processed using LabView and MATLAB algorithms. Essentially, a FFT was applied on each acquired signal, and the amplitude of the driving frequency of each scan was evaluated and stored in such a way that an air-coupled C-scan imaging of the inspected sample could be evaluated. A 5 kHz (holey structure 1 design frequency) 2.5 V positive square wave was used to excite the structures, by means of a waveform generator used in conjunction with a 50× power amplifier to provide more energy to the structures. Figure 11a illustrates the air-coupled C-scan imaging of the CFRP impacted plate, showing that the vibrational amplitude of the sample was much higher around the impact zone. Furthermore, a 3D scan imaging of the response was also evaluated (Figure 11b) to provide clearer damage localisation. Figure 13 shows the ultrasonic response for the inspection performed on the corroded plate, where stress-corrosion micro-cracks are clearly highlighted by an increase in the amplitude of the driving frequency (Figure 13b). Air-coupled inspections were also conducted on the aluminium plate without the metamaterial, and the amplitude response is shown in Figure 12. Results from the air-coupled inspection conducted without the metamaterials partially evaluated the damaged region, but with low resolution and a low signal-to-noise ratio, thus resulting in poor sensitivity of the inspection. In addition, the amplitude peaks of the damaged region were much higher in the investigations conducted with the proposed holey-structured metamaterial, as revealed by the colour scale in Figure 13, which assesses the resolving performance of the proposed metamaterial-base structure for improving damage imaging. The working principle of these results relies on the interaction between low-frequency waves (i.e., wavelength larger than the size of the metamaterial) and a subwavelength structure, thus resulting in a perturbating system. As a wave propagates through holey-structured metamaterial, a superposition of three physical effects occurs, i.e., the above-mentioned Fabry–Perot resonance inside the structure, elastic-surface modes and coherent scattering due to the periodicity of the holes contained in the metamaterial. Indeed, at the Fabry–Perot resonance frequency, the coupling of the arising scattered waves and the hole periodicity increase the amplitude of the propagating wave [33,42]. The proposed holey-structured metamaterial behaved as a passive sound filter to select frequency components from the incoming wave to the outgoing wave. These effects rely on the fact that a high-frequency component with wavelength λ<2d (i.e., hole length L=λ⁄2 for the Fabry–Perot resonance mode condition) propagates through the cavities more efficiently. As shown in Figure 11, Figure 12 and Figure 13, the amplitude frequency components concentrated on the damage region is related to an increase in the degradation of the local material properties (i.e., local stiffness), and can be filtered for clear damage evaluation. Finally, results have also shown the capability of the proposed metamaterial to locate both surface and subsurface damages. However, the defective area must be located at a maximum distance of one wavelength from the sample surface, and this can be controlled by adjusting the excitation frequency. In this scenario, the metamaterial’s geometrical parameters must be adapted to the required wavelength, according with the above-described Fabry–Perot resonance theory. The transmission test results in Figure 10 demonstrate the suitability of the proposed structures for both the acoustic (f < 20 kHz) and ultrasound frequency range (f > 20 kHz).

## 5. Conclusions

Air-coupled ultrasonic inspections in the nondestructive evaluation of materials have represented a valid alternative for damage detection in materials where inspections involving contact are not feasible. However, the acoustic mismatch between air and solids plays a challenging role, as transmission of energy can be unacceptable for nonlinear distortion due to damage. In this regard, a design of a deep-subwavelength holey-structured metamaterial lens was proposed to improve wave propagation in the context of nonlinear inspection. The geometrical parameters of the proposed structure were optimized to couple the decaying evanescent waves with the Fabry–Perot resonance mode of the lens, resulting in an enhancement of the evanescent wave that propagated through the cavities inside the lens. Transmission test results showed an increased amplitude of the design frequency of the lens of up to 254% compared with free propagation. Finally, noncontact air-coupled inspections were performed on a stress-corrosion cracked sample and a barely visible impact damaged (BVID) plate. The results confirmed the effectiveness of the metamaterial lens in the context of acousto-ultrasonic imaging, thus demonstrating the potential of the proposed method of deep-subwavelength acousto-ultrasonic imaging of various damages and micro-cracks in air-coupled inspections.

## Figures and Tables

**Table 1:** Acoustic impedances for various media.

Medium	Acoustic Impedance [MRayls]
**Aluminium**	17.1
**Stainless Steel**	46.6
**CFRP**	5.5–6.2
**Water (20 °C)**	1.49
**Air (20 °C)**	0.00043

**Table 2:** Main characteristics of the holey structures.

Holey Structure	Hole Size *d* [mm]	Wall Thickness *w* [mm]	Length *L* [mm]	Design Frequency [kHz]
S1	4.9	4.9	34.3	5
S2	1.715	1.715	8.575	20
S3	1.143	1.143	5.716	30
S4	0.8575	0.8575	4.288	40

## Data Availability

Not applicable.
